# Glances at the Hospitals

**Published:** 1902-02-01

**Authors:** 


### GLANCES AT THE HOSPITALS.

### THE FEVER HOSPITAL, DUBLIN.

Including a parliamentary grant of £2,500 the income was £15,327, and the receipts were £16,403. The Bank account was overdrawn by £4,186. The total number of patients admitted was 2,508. Of this total, five diseases—enteric fever, scarlatina, measles, pneumonia, and diphtheria—account for 1,892, and of this enteric fever was responsible for 404. There were 119 cases of diphtheria; and the physicians' report states that "as we become more familiar with the disease it becomes increasingly apparent that there is some peculiar relationship between diphtheria and scarlatina"; and they add that on "more than one occasion a case has come in from a private house with scarlatina, to be followed two or three days later by a case from the same family of diphtheria without sign or symptom of scarlatina. Convalescents from one disease have developed the other without any possible source of infection having been discovered." It is beyond question that diphtheria and scarlatina are sometimes concurrent in the same patient, and so are diphtheria and measles and diphtheria and small-pox; but whether this association amounts to relationship is another matter, and it is one which considerable research and observation would be needed to substantiate. Referring more particularly to the excellent tables printed on page 40, *et seq.*, we notice that of the 404 cases of enteric fever 35 died, being a percentage of 8·66. Of the 323 cases of scarlatina 31 died, being a percentage of 9·59. Of the 119 cases of diphtheria 14 died, being a percentage of 11·47. The Visiting Committee draw attention to the fact that no nurses' home is attached to the fever hospital. This is a serious deficiency, and that it should be allowed to continue is hardly creditable to the population of Dublin. It goes without saying that during their hours off duty the nurses should be away from contamination with the air of the wards.

ASHTON-UNDER-LYNE INFIRMARY.

The total general income was £3,669, and the total expenditure was £3,540. We are pleased to note that the workpeople's subscriptions increased from £611 to £648. The daily average number of beds occupied was 65, the cost per bed was £49, the cost per week per patient was 18s. 10d., and the average stay in the infirmary was 38 days. The latter we look upon as very high. A page of letterpress from the honorary medical officers is inserted, and from this we learn that 1,606 cases were treated during the year, and that of this number 616 were in-patients. There were 54 deaths, 13 being due to severe accidents. The number of operations performed was 311. Regarding the latter, it is stated that "the results have, on the whole, proved highly gratifying." The house surgeon's report shows the numbers treated for the various diseases named, the numbers cured, and the numbers died. We failed to notice any operation table; and we venture to suggest that in subsequent reports this table should be supplied, and that the medical statistics should be together and not separated by financial statements.

ROYAL HOSPITAL FOR SICK CHILDREN, GLASGOW.

The ordinary income was £3,297, and the ordinary expenditure was £5,182, showing a deficiency of £1,885, which was carried to the capital account. The extraordinary income, however, amounted to £4,633. At the beginning of the year there were 72 patients in residence, and 714 were admitted during the year, making a total of 786. The average daily number resident was 66, and the average stay in the wards was 31 days. The total cost of each patient treated was £5 3s., and the daily cost of each was 3s. 3d. There are tables showing the nature of the cases admitted, and the nature of the operations performed in the hospital, but no results are given. There is, however, a table showing the causes of death, and by wading through this we can arrive at some knowledge of the results. For instance, we notice that five cases of enteric fever were admitted, and as this disease does not appear in the death table, we assume that all the five cases recovered, there were also 16 cases of tubercular meningitis, and the table shows that 10 died of this disease, but we know nothing as to the other six. We hope that the next report will supply clearer information.

NORFOLK AND NORWICH HOSPITAL.

The total income was £9,755, and the total expenditure was £10,225. The daily average number of in-patients was 147, and the average stay in the hospital was the long period of 32 days. The average cost of each bed occupied was £63 8s.; the cost of each patient per week was 24s. On January 1st the hospital contained 126 patients, and 1,542 were admitted during the year, making a total of 1,668. Of these 755 were discharged cured, 346 relieved, 163 were made out-patients, 38 were discharged for various reasons, 98 died, and 142 remained under treatment. It should be noted that 14 of the deaths occurred within 24 hours of admission. At the out-patients' department 6,381 patients were treated. The medical and surgical tables are compiled with much care. Every disease is set forth, and columns show the age, sex, habitation, and result of the treatment. For instance, 40 patients with phthisis were in the hospital during the year. Of these 24 came from the city, and 16 from the country. Of the total 2 were cured, 25 relieved, 3 made out-patients, 2 discharged for other reasons, 1 died, and 7 remained under treatment. There were 700 operations performed during the year, and the table gives the disease but not the results, so that it is necessary to turn to the general tables and note the number dying of any particular surgical disease. We hope this will be remedied in next year's report, and that a table will be inserted showing the nature of the anæsthetic used.

ROYAL HALIFAX INFIRMARY.

The receipts amounted to £8,086 and the expenditure to £9,542, there being a balance of £1,456 due to the bank; but this adverse balance remained from the previous year, and no increase to this balance was made during the year under notice. The governors say that if the Infirmary is to be maintained at its present efficiency still more support must be received from the public. In the meantime it is most gratifying to report that the workpeople's contributions amounted to £2,767; and as long as this is kept up the workpeople may feel sure that other help will be forthcoming. The total number of beds is 150, and 1,587 cases were treated during the year. The average number of in-patients was 111; the cost of each was £4 8s., and the average stay in the hospital was 25 days. The cost per bed occupied was £62 18s. The average cost of food per head per week for the entire household and patients was 4s. 9d. The medical and surgical tables are prepared in such a careful way that all essential information is conveyed.

WORCESTERSHIRE GENERAL INFIRMARY.

The income for the year was £3,981, and there was an adverse balance of £2,656. As a consequence of this, the governors sold £3,000 of their funded property. The funds in hand are still large; but the step is never a pleasant one for a board to take. It is stated that the contributions from the public show a falling off, and so do donations and collections, while the demands on the benefits of the institution show no decrease. The amount of the annual subscriptions was only £1,296, and it is possible that an appeal to the workpeople themselves might result in an increase in their contributions. There were 91 patients in residence on January 1st, and 1,069 were admitted during the year. Of these 678 were cured, 276 were relieved, 46 not improved, 89 died, and 71 remained under treatment. The daily average number resident was 95, and the cost per bed was £49 3s. The medical and surgical tables show the numbers treated for various diseases, and there are columns showing the results, but the operations' table merely gives the numbers operated on, and the anæsthetic used is not stated.

